# Prediction of neurosurgical intervention after mild traumatic brain injury using the national trauma data bank

**DOI:** 10.1186/s13017-015-0017-6

**Published:** 2015-06-06

**Authors:** Timothy E. Sweeney, Arghavan Salles, Odette A. Harris, David A. Spain, Kristan L. Staudenmayer

**Affiliations:** Department of Surgery, Stanford University Medical Center, 300 Pasteur Drive, Stanford, CA 94305 USA; Department of Neurosurgery, Stanford University Medical Center, 300 Pasteur Drive, Stanford, CA 94305 USA

**Keywords:** Traumatic brain injury, National trauma data bank, Neurosurgery

## Abstract

**Introduction:**

Patients with mild traumatic brain injury (TBI) as defined by an admission Glasgow Coma Score (GCS) of 14–15 often do not require neurosurgical interventions, but which patients will go on to require neurosurgical care has been difficult to predict. We hypothesized that injury patterns would be associated with need for eventual neurosurgical intervention in mild TBI.

**Methods:**

The National Trauma Databank (2007–2012) was queried for patients with blunt injury and a diagnosis of TBI with an emergency department GCS of 14–15. Patients were stratified by age and injury type. Multiple logistic regression for neurosurgical intervention was run with patient demographics, physiologic variables, and injury diagnoses as dependent variables.

**Results:**

The study included 50,496 patients, with an overall 8.8 % rate of neurosurgical intervention. Neurosurgical intervention rates varied markedly according to injury type, and were only correlated with age for patients with epidural and subdural hemorrhage. In multiple logistic regression, TBI diagnoses were predictive of need for neurosurgical interventions; moreover, after controlling for injury type and severity score, age was not significantly associated with requiring neurosurgical intervention.

**Conclusions:**

We found that in mild TBI, injury pattern is associated with eventual need for neurosurgical intervention. Patients with cerebral contusion or subarachnoid hemorrhage are much less likely to require neurosurgical intervention, and the effects of age are not significant after controlling for other patient factors. Prospective studies should validate this finding so that treatment guidelines can be updated to better allocate ICU resources.

## Background

Traumatic brain injury (TBI) accounts for >1.3 million Emergency Department (ED) visits and >750,000 hospitalizations each year [1]. A large number of TBI patients present with a Glasgow Coma Score (GCS) of 14–15 and do not ultimately require an intervention for their injuries. Which patients ultimately require intervention has been difficult to predict, and there are no clear consensus guidelines for treatment of this patient subset (in contrast to the extensive guidelines for severe TBI [2]). For instance, the American College of Emergency Physicians’ Mild TBI policy from 2008 offers recommendations on discharging patients without intracranial hemorrhage, but patients with GCS 14–15 and positive CT findings are not discussed [3]. Many hospital guidelines currently suggest that all patients with intracranial hemorrhage of any severity be observed in the intensive care unit (ICU) due to risk of decompensation and possible need for intervention. However, these recommendations are not evidence-based [4]. The lack of clear consensus for treatment of mild TBI leads to a wide variability in clinical practice, with initial ICU admission rates ranging from 50–97 % for patients with a GCS of 15 and traumatic intracranial hemorrhage [5].

Several prior studies have been published examining what factors contribute to decompensation in patients with mild TBI [5–9]. Factors that are typically part of the resulting models include older age, high-volume intracranial hemorrhage and/or midline shift, anticoagulant therapy, and worsening injury. However, these studies have mostly been from single-center or regional databases and thus may not be generalizable.

We hypothesized that injury type would be associated with deterioration for patients who present with isolated mild TBI. To explore this, we evaluated the need for a neurosurgical procedure in patients who presented with isolated mild TBI using the National Trauma Data Bank.

## Methods

We used the National Trauma Data Bank (NTDB) from 2007 to 2012. The year 2012 is the most recent year for which data are available. Patients were included if they were adults (> = 18 years of age) with an International Classification of Diseases, Ninth Revision, Clinical Modification (ICD-9-CM) diagnosis of intracranial injury (851.0–854.9), were admitted to the hospital, and had an ED total GCS of 14–15. Skull fracture diagnoses (800–801.9, 803–804.9) were not included as the ICD-9-CM diagnosis codes do not distinguish which type of intracranial lesion is present. Also, open fractures present an indication for operative intervention making determination of intracranial injury progression difficult. Patients were also excluded if they had sustained a penetrating mechanism of injury or if they had an abbreviated injury scale (AIS) severity score of >1 in any body region other than the head. Patients with missing data on ED vital signs were excluded.

Head injuries were binned into six categories by ICD-9-CM code: isolated cerebral laceration or contusion (851.0–851.9), isolated subarachnoid hemorrhage (852.0–852.1), isolated subdural hemorrhage (852.2-852.3), isolated epidural hematoma (852.4–852.5), and unspecified (853–854.9). Patients with more than one of the above types of TBI were categorized only as ‘multiple TBI injuries.’

Whether a neurosurgical intervention was performed was also determined. Neurosurgical intervention was defined as having either an operative neurosurgical procedure or placement of a neuromonitoring device (e.g., Camino bolt or endoventricular drainage catheter). Surgeries and placement of catheters were identified using ICD-9-CM procedure codes of 01–02.

Injury severity score (ISS) calculated from the AIS severity codes was evaluated in this model. ISS is calculated as the sum of the square of the top three AIS severity scores (by body region). Since here we only included patients whose non-head AIS severity scores were < =1, the maximum ISS any patient can receive is the square of the AIS head severity score plus two. We thus discretized ISS from 0–6, 7–11, 12–18, 19–27, and >27, with the assumption that increasing ISS is solely due to worsening severity of head injury.

In the NTDB, coagulopathy is defined as any condition that places the patient at risk for bleeding in which there is a problem with the body’s blood clotting process (e.g., vitamin K deficiency, hemophilia, thrombocytopenia, chronic anticoagulation therapy with Coumadin, Plavix, or similar medications.) This does not include patients on chronic aspirin therapy. More granular information about exact anticoagulant drugs, dosages, etc., are not available. The presence of coagulopathy was thus coded as a binary variable.

Multiple logistic regression was used to predict the need for neurosurgical intervention. Dependent variables included in the analysis were age; presence of coagulopathy; ED vital signs; injury severity score (ISS) coded as described above; head injury type (coded in a binary form according to the categories defined above). The same model was also run as a mixed-effects model with different hospital facilities as the random-effects variable to control for center effect.

All statistical analyses were carried out in the R language for statistical computing version 3.0.1. Comparisons between two cases were done with two-sided Student’s t-tests. Significance levels were set at P < 0.01 unless otherwise stated.

## Results

The NTDB 2007–2012 dataset contained 1.3 million cases of traumatic brain injury. After applying inclusion and exclusion criteria, there were a total of 50,496 patients (Table 1). Isolated subdural hemorrhages (SDH) were the most common injury pattern (N = 18,784, 37 %), and subarachnoid hemorrhages were the second most common isolated injury (N = 13,191, 26 %) (Table 1). Most patients were treated at a Level I or II trauma center (N = 34,961, 69.2 %), and the majority of patients were admitted directly to the intensive care unit (N = 29,043, 58 %). The overall rate of neurosurgical intervention was 8.8 %.

**Table 1:** Patient demographics, injury patterns, and disposition. For discrete variables, percentages are calculated by dividing by total patients (not by the number of patients with the given variable)

	All patients		No neurosurgical intervention		Neurosurgical intervention	
	N or mean	% or SD	N or mean	% or SD	N or mean	% or SD
Total Included Patients	50,496	100 %	46,022	91.2 %	4,474	8.8 %
Demographics						
Male gender (N, %)	30386	60.2	27407	59.6	2979	66.6
Age (years) (mean, SD)	60.6	20.5	60.2	20.7	65.2***	18.3
Physiology						
ED GCS (mean, SD)	14.8	0.4	14.8	0.4	14.7***	0.4
ED SBP (mean, SD)	144.4	26.4	144.1	26.4	147.6***	26.6
ED Pulse (mean, SD)	85.3	18	85.6	18	81.7***	18
ED RR (mean, SD)	18.1	3.7	18.2	3.8	17.9***	3.4
Injury Characteristics						
ISS at discharge (mean, SD)	13.7	6.5	13.1	6.1	19.7***	6.7
Traumatic Brain Injury Patterns						
Isolated Contusion (N, %)	5636	11.2 %	5497	11.9 %	139	3.1 %
Isolated SAH (N, %)	13191	26.1 %	12994	28.2 %	197	4.4 %
Isolated SDH (N, %)	18784	37.2 %	15807	34.3 %	2977	66.5 %
Isolated EH (N, %)	901	1.8 %	742	1.6 %	159	3.6 %
Multiple Injury Types (N, %)	11984	23.7 %	10982	23.9 %	1002	22.4 %
Comorbidities						
Total comorbidities (mean, SD)	0.9	1.1	0.9	1.1	0.9	1.2
Presence of Coagulopathy (N, %)	2340	4.6 %	2061	4.5 %	279	6.2 %
ACS Trauma Center Level						
NA/ Unverified (N, %)	14713	29.1 %	13214	28.7 %	1499	33.5 %
Level IV (N, %)	20	0 %	19	0 %	1	0 %
Level III (N, %)	802	1.6 %	730	1.6 %	72	1.6 %
Level II (N, %)	13200	26.1 %	12110	26.3 %	1090	24.4 %
Level I (N, %)	21761	43.1 %	19949	43.3 %	1812	40.5 %
ED Disposition						
Observation unit (N, %)	827	1.6 %	818	1.8 %	9	0.2 %
Floor bed (N, %)	13329	26.4 %	12756	27.7 %	573	12.8 %
Telemetry/step-down unit (N, %)	5292	10.5 %	5122	11.1 %	170	3.8 %
Intensive Care Unit (ICU) (N, %)	29043	57.5 %	26580	57.8 %	2463	55.1 %
Operating Room (N, %)	2005	4 %	746	1.6 %	1259	28.1 %
Outcomes						
LOS (mean days, SD)	5.4	6.5	4.8	5.5	11.2***	11.2
Expired during Admission (N, %)	1594	3.2 %	1141	2.5 %	453	10.1 %

*N* Number, *SD* Standard Deviation, *ISS* Injury Severity Score, *ED* Emergency Department, *GCS* Glasgow Coma Score, *SBP* Systolic Blood Pressure, *RR* Respiratory Rate, *SAH* Subarachnoid Hemorrhage, *SDH* Subdural Hemorrhage, *ED* Epidural Hemorrhage, *LOS* Length of Stay

*** P < 0.0001; Student’s *t*-test for differences of continuous measures between “No Neurosurgical Intervention” and “Neurosurgical Intervention” groups

**Fig. 1:**
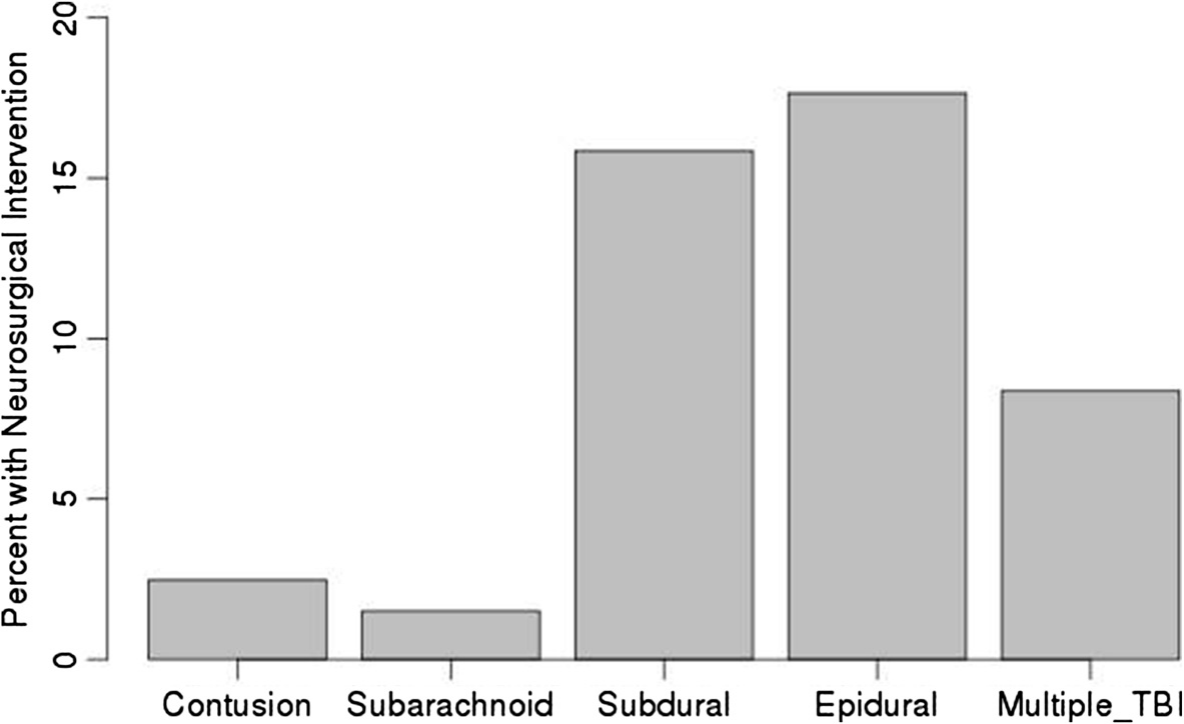
Percentage of patients requiring neurosurgical intervention according to injury subtype

Patients who underwent neurosurgical intervention were overall older (mean 65 vs 60 years, P < 0.0001), had higher ISS (mean 19.7 vs 13.1, P < 0.0001), and had a slightly lower ED GCS (14.7 vs. 14.8, P < 0.0001) compared to those who did not. Isolated epidural hemorrhages were most frequently associated with neurosurgical procedures (18 %), followed by isolated subdural hemorrhages (16 %) and multiple injury types (8 %) (Fig. 1). Isolated subarachnoid hemorrhages and contusions were infrequently associated with need for neurosurgical procedures (1.5 and 2.5 %, respectively).

**Fig. 2:**
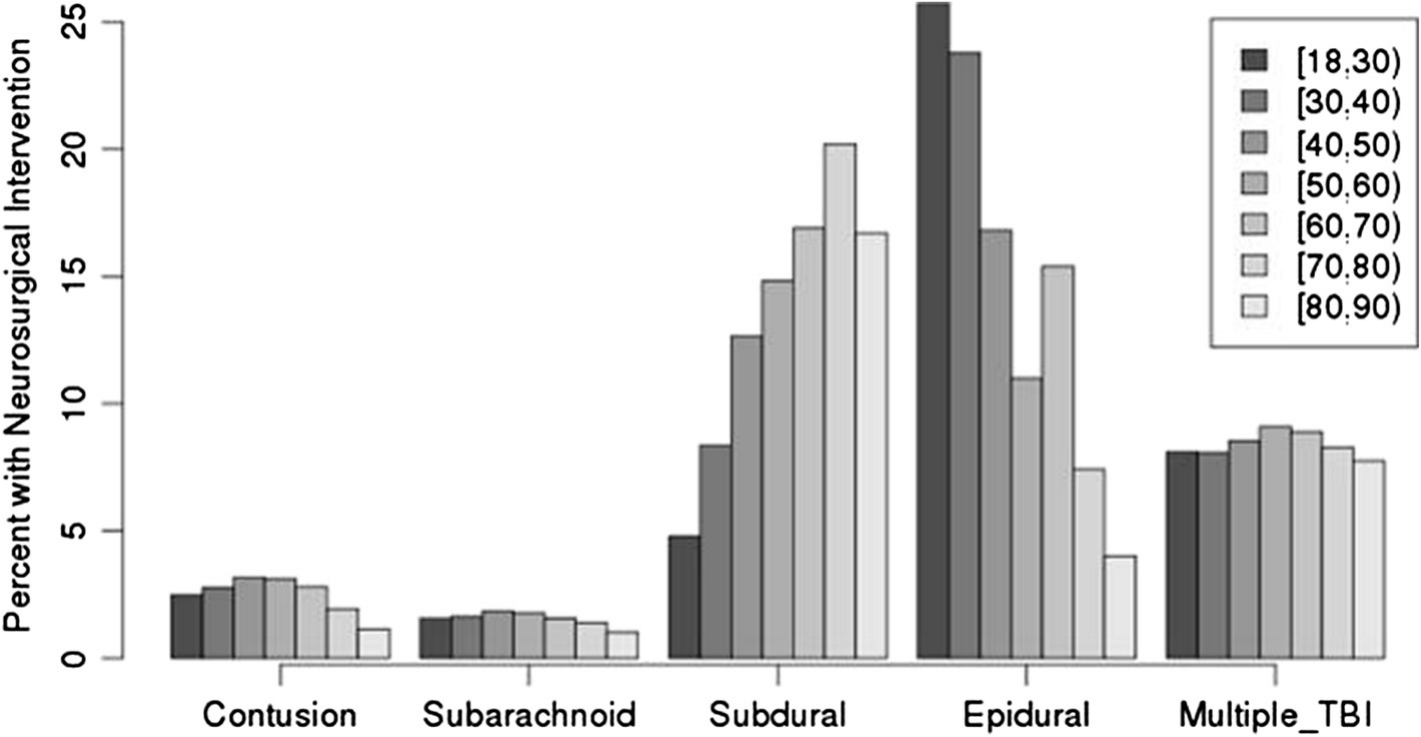
Percentage of patients requiring neurosurgical intervention, stratified by both injury subtype and age decile

We found that patients with SDH who underwent neurosurgical procedures were older than those who did not (70.2 vs 65.7 years, P < 0.0001), whereas patients with EDH who underwent neurosurgical procedures were younger (37 vs 48 years, P < 0.0001) (Table 2). Age was not a significant factor for the other injury types. On breaking out interventions by age group, there was a positive correlation with age and neurosurgical intervention rates for the SDH cohort, but a negative correlation for the EDH cohort (Fig. 2).

**Table 2:** Age and neurosurgical intervention for different injury patterns

Age (years)	No neurosurgical intervention	Neurosurgical intervention	*P*-value
	Mean ± SD	Mean ± SD	
Isolated Contusion	51.4 ± 21.8	48.5 ± 19.0	0.08
Isolated SAH	58.7 ± 20.1	56.1 ± 19.4	0.07
Isolated SDH	65.7 ± 19.1	70.2 ± 14.7	<0.0001
Isolated EH	48.0 ± 22.3	37.0 ± 17.2	<0.0001
Multiple Injury Types	59.4 ± 20.7	59.0 ± 20.0	0.56

*P*-values from Student’s *t*-test for differences of continuous measures between “No Neurosurgical Intervention” and “Neurosurgical Intervention” groups

*SD* Standard Deviation, *SAH* Subarachnoid Hemorrhage, *SDH* Subdural Hemorrhage, *ED* Epidural Hemorrhage

**Fig. 3:**
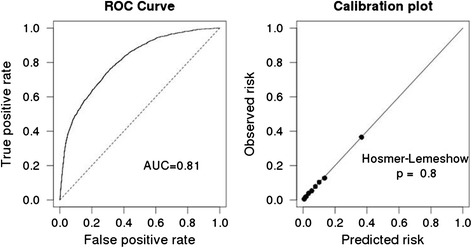
Performance evaluation of the multiple logistic regression model on a held-out test set (n = 17,169). **a**. Receiver Operating Characteristic curve for the test set, area under the curve (AUC) = 0.81. **b**. Calibration plot; Hosmer-Lemeshow P = 0.8

The dataset was next randomly split into a 2/3 training set and a 1/3 test set. A multiple logistic regression model for predicting neurosurgical intervention was created from the training set (N = 33,327) (Table 3). After adjusting for injury severity, age, coagulopathy, and ED vital signs, injury pattern was strongly associated with need for neurosurgical intervention. Age was not significantly associated with need for neurosurgical intervention. The odds ratio for need for neurosurgical intervention for patients with an EDH vs. contusion was 6.4 (95 % CI 4.1–9.9). When applied to the held-out test set (N = 17,169), this model had good performance with an area under the receiver operator characteristics (ROC) curve for prediction of neurosurgery of 0.81 (Fig. 3a). It also showed excellent calibration (Hosmer-Lemeshow P = 0.8) (Fig. 3b). Interestingly, the calibration plot shows that our model’s highest-risk decile has a modest expected (and observed) rate of neurosurgery of 38 %; the model is more effective at identifying very low-risk patients (lowest decile expected 0.5 % rate of neurosurgery). A mixed-effects model for which facility was used as a random effect was also performed; it showed no qualitative change in coefficients or significance (results not shown).

**Table 3:** Adjusted odds ratios for neurosurgical procedures. Multiple logistic regression run on 2/3 training set (n = 33,327)

	Odds ratio (95 % CI)		*P*-value
(Intercept)	0.0893	(0.0099 – 0.78)	0.03
Age (years)	1.002	(0.999 – 1.01)	0.18
Anticoagulation Disorder	0.853	(0.66 – 1.09)	0.21
ED GCS	0.894	(0.781 – 1.03)	0.11
ED Systolic Blood Pressure	1.004	(1.002 – 1.01)	<0.001
ED Pulse	0.99	(0.986 – 0.993)	<0.0001
ED Respiratory Rate	0.962	(0.944 – 0.98)	<0.0001
ISS Category (vs. ISS 0–6)			
ISS 7-11	2.35	(1.44 – 4.09)	<0.01
ISS 12-18	3.37	(2.06 – 5.86)	<0.0001
ISS 19-27	18.9	(11.6 – 33)	<0.0001
ISS >27	7.01	(3.79 – 13.4)	<0.0001
Injury Category (vs. Contusion)			
Isolated SAH	0.95	(0.64 – 1.41)	0.79
Isolated SDH	4.9	(3.61 – 6.84)	<0.0001
Isolated EDH	6.42	(4.15 – 9.97)	<0.0001
Multiple Injury Types	2.34	(1.7 – 3.29)	<0.0001

*CI* Confidence Interval, *ISS* Injury Severity Score, *SD* Standard Deviation, *SAH* Subarachnoid Hemorrhage, *SDH* Subdural Hemorrhage, *ED* Epidural Hemorrhage

## Discussion

Traumatic brain injuries are an increasing source of emergency department visits and morbidity in the United States [10]. Mild traumatic brain injuries (those with a presentation GCS 14–15) with associated intracranial hemorrhage often present a clinical challenge, as acute decompensation in this cohort is rare but serious. As it has not been possible to predict which mild injuries will progress, many centers have policies of admitting all mild head injuries to a critical care or stepdown setting. This likely is beneficial for the small sub-group of patients who progress, but is associated with high costs and resource utilization.

Here we show that injury pattern may be important in determining which patients are at higher risk for ultimately requiring a neurosurgical intervention. Injury pattern is strongly associated with need for future neurosurgical procedures. In general, patients with SDH represent the largest number of interventions, and in this group older age is correlated with greater requirement for neurosurgical intervention. Previous reports have found that age is independently associated with higher associated rates of decompensation [7–9, 11], but this did not bear out when controlling for both injury type and physiologic variables. The finding that age is associated with need for intervention is likely due to the fact that patients with SDH are older, and there tend to be more of them than the other injury patterns (Table 2). These findings are consistent with the classic teaching of risk for SDH in elderly patients due age-related reductions in intracranial mass resulting in strain on bridging veins.

Epidural hemorrhages were far more infrequent (~2 %) than subdural hemorrhages but had the highest rates of neurosurgical intervention (21 %). Of those with epidural hemorrhages, younger patients had the highest rates of need for intervention. These findings are not particularly surprising given the fact that epidural hemorrhages often represent arterial bleeding and therefore have a higher risk of mass effect. The presence of multiple TBI patterns was also associated with higher rate of intervention. It may be that in these cases, higher force of impact resulted in multiple types of injuries and therefore these patients should be carefully monitored for worsening of their condition.

In contrast to SDH, SAH and contusions were much less often associated with the need for neurosurgical intervention. This is consistent with anecdotal reports of small injuries with normal GCS not requiring advanced care. That said, while a 1–2 % rate of neurosurgical intervention may seem small, it still represents hundreds of patients who ultimately required advanced care. Patients with SAH and contusion were only part of the broader cohort with a very low predicted need for neurosurgical intervention. Further prospective studies will need to determine whether there are other characteristics or early signs that can predict which low-risk TBI patients with a GCS of 14–15 will deteriorate. If our findings are tested prospectively and characteristics that predict deterioration are validated, patients without these types of injuries may represent candidates for a non-monitored setting. This would have a large impact on resources and costs as together these injuries comprise 36 % of the TBI population in trauma centers in the United States.

The findings from this study are consistent with previous reports. There is evidence from single-center studies that type of head injury (e.g., subdural hemorrhage vs. epidural hematoma vs. contusion) might be associated with progression of injury [6, 12]. However, these are both single-center studies with small numbers. Other studies have tried to make prediction models of outcomes after minor head trauma [6, 7, 9, 13–17]. In particular, Nishijima et al. found that a rule with four parameters (abnormal mental status (GCS < 15), non-isolated head injury, age > 65 years, and swelling/shift on CT) was 98 % sensitive and 50 % specific for predicting need for any “critical care intervention.” [11] However, the study included patients who had injuries other than TBI and the definition for “critical care intervention” included need for blood transfusion and central line placement. This does not help to answer the question of whether we can predict whether a mild isolated TBI will decompensate. In contrast, in our study we chose to evaluate isolated head trauma in order to prevent confounding that results from how non-TBI injuries may impact the course of a head injury.

Another factor thought to be associated with worsening of head injuries is pre-existing coagulopathy. In our multiple logistic regression model for predicting need for neurosurgical intervention, preexisting coagulopathy was not found to be a significant factor. This may be due to the fact that this variable may not be reliably recorded in trauma registries. Previous studies published from smaller, more granular trauma registries have shown that coagulopathy does predict decompensation [7, 8, 18].

This study has several limitations. First, this is a retrospective registry study that is subject to selection bias. In addition, we excluded all samples missing required data which relies on a missing-at-random assumption. Second, the NTDB does not capture neuro-critical care such as hyperosmolar therapy and hourly neurologic checks. Third, the NTDB does not capture information about the volume of intracranial hemorrhage, which may prove to be predictive. Finally, we did not model what happens in patients who sustain multi-system injury.

Despite these limitations, this study is the first to show an association between injury pattern and need for neurosurgical intervention in a national database. Overall, this study shows that in isolated blunt mild traumatic brain injury, SDH and EDH are associated with the highest rates of need for neurosurgical intervention, and that contusions and SAH are associated with low risks. Older age is associated with increasing rates of neurosurgical intervention after isolated SDH but is not a general predictor of need for neurosurgery in all types of injury. The accuracy of the model at predicting which patients are very unlikely to proceed to neurosurgical intervention suggests that these patients may not require higher levels of care (such as mandatory admission to an intensive care unit), albeit with a caveat that a 1-2 % rate of neurosurgical intervention is not negligible. Improved prediction of the need for intervention can allow us to better match resource with patient need, saving lives and improving allocation of resources. Further prospective studies of outcomes after mild TBI should include injury type as a predictor so that these issues can be further elucidated.
